# Serum exosomal miR-122-5p, GAS, and PGR in the non-invasive diagnosis of CAG

**DOI:** 10.1515/med-2021-0342

**Published:** 2021-09-08

**Authors:** Naihua Liu, Yuancheng Huang, Fengbin Liu, Hong Liu

**Affiliations:** Department of Traditional Chinese Medicine, The First Affiliated Hospital of Guangdong Pharmaceutical University, Guangzhou 510080, Guangdong, People’s Republic of China; Department of Oncology, Scientific Research Center, The First Affiliated Hospital of Guangdong Pharmaceutical University, Guangzhou 510080, Guangdong, People’s Republic of China; Department of Gastroenterology, Research Center for Engineering Techniques of Microbiota-Targeted Therapies of Guangdong Province, Guangzhou 510080, Guangdong, People’s Republic of China; Department of Chinese Internal Medicine, Lingnan Medical Research Center of Guangzhou University of Chinese Medicine, Guangzhou 510405, Guangdong Province, People’s Republic of China; Department of Spleen Disease and Gastropathy, The First Affiliated Hospital of Guangzhou University of Chinese Medicine, No. 16 Jichang Road, Guangzhou 510405, Guangdong Province, China; Department of Chinese Internal Medicine, Lingnan Medical Research Center of Guangzhou University of Chinese Medicine, No. 12 Jichang Road, Guangzhou 510405, Guangdong Province, People’s Republic of China; Department of Traditional Chinese Medicine, The First Affiliated Hospital of Guangdong Pharmaceutical University, Gonghexiheng Street 1, Guangzhou 510080, Guangdong, People’s Republic of China

**Keywords:** serum exosomes, hsa-miR-122-5p, non-invasive diagnosis, chronic atrophic gastritis

## Abstract

**Objective:**

The aim of this study was to integrate the serum exosomal miRNA miR-122-5p with canonical serological biomarkers for the non-invasive screening of chronic atrophic gastritis (CAG) patients.

**Methods:**

miR-122-5p and U6 were amplified by the quantitative reverse transcription polymerase chain reaction (RT-qPCR), gastrin (GAS), pepsinogen I (PG-I), and PG-II and were measured by ELISA. The area under the receiver operating characteristic (ROC) curves and their correlation were analyzed.

**Results:**

In the present study, GAS level and PG-I/PG-II ratio (PGR) were increased in CAG group, but there was no significant difference in PG-I or PG-II levels between CAG group and chronic non-atrophic gastritis (CNAG) group. Only GAS level and PG-I/PG-II ratio were significantly correlated with atrophy, and not any other clinicopathologic factors. Expression of hsa-miR-122-5p positively correlated with GAS level, PG-I level, and PGR, while it negatively correlated with PG-II level; however, none of them had significant difference. The combination of GAS, PGR, and hsa-miR-122-5p presented as a better model for non-invasive screening of CAG compared to others.

**Conclusion:**

These results suggested that serum exosomal hsa-miR-122-5p combined with GAS and PGR would elevate accuracy and specificity in non-invasive screening of CAG.

## 1 Introduction

Chronic atrophic gastritis (CAG) is a precancerous, wild-spread gastrointestinal disease. The prevalence of CAG is 10% in 20–50 years old population, and more than 50% in 51–65 years old population [1]. In China, the proportion of CAG is different between regions, but CAG is commonly and highly correlated with the incidence of gastric cancer [2]. Clinically, the diagnosis and staging of CAG mainly depended on the presence of chronic inflammatory cells established by endoscopic and histological examination, including lymphocytes and plasma cells that expanded in lamina propria, and the disappearance of the normal glands [3,4,5]. Up to date, diagnostic methods of CAG are usually invasive, which are hardly accepted by the patients [2]. Fortunately, accumulating serological biomarkers were highlighted in CAG diagnosis. It is reported that gastrin (GAS), pepsinogen I (PG-I), or pepsinogen II (PG-II) might be helpful for non-invasive diagnosis of atrophic gastritis [2,6]. Besides, previous study showed that the sensitivity and specificity of a panel test (G-17, PG, and anti-*Helicobacter pylori*) was 74.7 and 95.6%, respectively [7].

Exosomes, a form of endosome-derived extracellular vesicle, transfer various proteins, lipids, and nucleic acids to play an important role in cell–cell communication process [8]. MicroRNAs (miRNAs) consist of approximately 21–25 nucleotides, which function to regulate translational repression or mRNA degradation [9]. miRNAs can be detected in blood, bound to protein complexes [10]. Exosome is an enclosed vesicle, which in turn provide a stable environment to protect miRNAs from RNase degradation [11]. Interestingly, serum exosomal miR-19b-3p and miR-106a-5p were suggested as diagnostic biomarkers for gastric cancer [12]. However, serum exosomal miRNAs tested for diagnosis of CAG patients remain largely elusive.

Our previous study first screened and compared the serum exosomal miRNA expression profile between CAG group and chronic non-atrophic gastritis (CNAG) group, and these previous results suggested that hsa-miR-122-5p has a potential diagnostic value for CAG [13]. Thus, in the present study, we further evaluated potential value of hsa-miR-122-5p combination with other serological markers on non-invasive diagnosis of CAG patients.

## 2 Materials and methods

### 2.1 Patients and samples

CNAG (*n* = 30 cases) and CAG (*n* = 30 cases) patients recruitment, serum samples collection, and the raw data of serum exosomal miRNA expression profile were shared from our previous study, and will be found here: https://doi.org/10.1186/s12885-019-5328-7 [13]. Briefly, all participants were recruited from 2016 to 2017 at the First Affiliated Hospital of Guangzhou University of Chinese Medicine in Guangzhou, China. They all confirmed that they were not carrying *Helicobacter pylori*. Then, these participants were divided into the CAG group and the CNAG group to receive physical examination, laboratory safety tests, gastroscopy, and biopsies. The CAG group was accompanied with or without intestinal metaplasia. The CNAG group was composed of health honors and patients who had mild or moderate superficial gastritis.

**Ethics approval and consent to participate:** The study was approved by the Ethics Committee of Guangzhou University of Traditional Chinese Medicine First Affiliated Hospital, and it was one part of our trial study (ChiCTR-IOR-16008027, http://www.chictr.org.cn/showproj.aspx?Proj=12924). All participants provided informed consent.

### 2.2 ELISA for serum GAS, PG-I, or PG-II concentrations

Enzyme-linked immunosorbent assay (ELISA) were performed using Human GAS/PG-I/PG-II Kit (Cusabio. China). Fifty microliter of serum was used to measure PG-I, PG-II, and gastrin-17 (G-17) concentrations according to the manufacturers’ instructions.

### 2.3 Statistical analysis

All statistical analyses were performed using the SPSS software (20.0 version), and the graphs were generated using GraphPad Prism 7.0 (GraphPad Software, San Diego, CA, USA). The difference among treatment groups were analyzed by one-way ANOVA and Student’s *t*-test. *p* < 0.05 considered as statistically significant.

## 3 Results

### 3.1 Serum GAS, PG-I, and PG-II level in CAG patients

**Figure 1:**
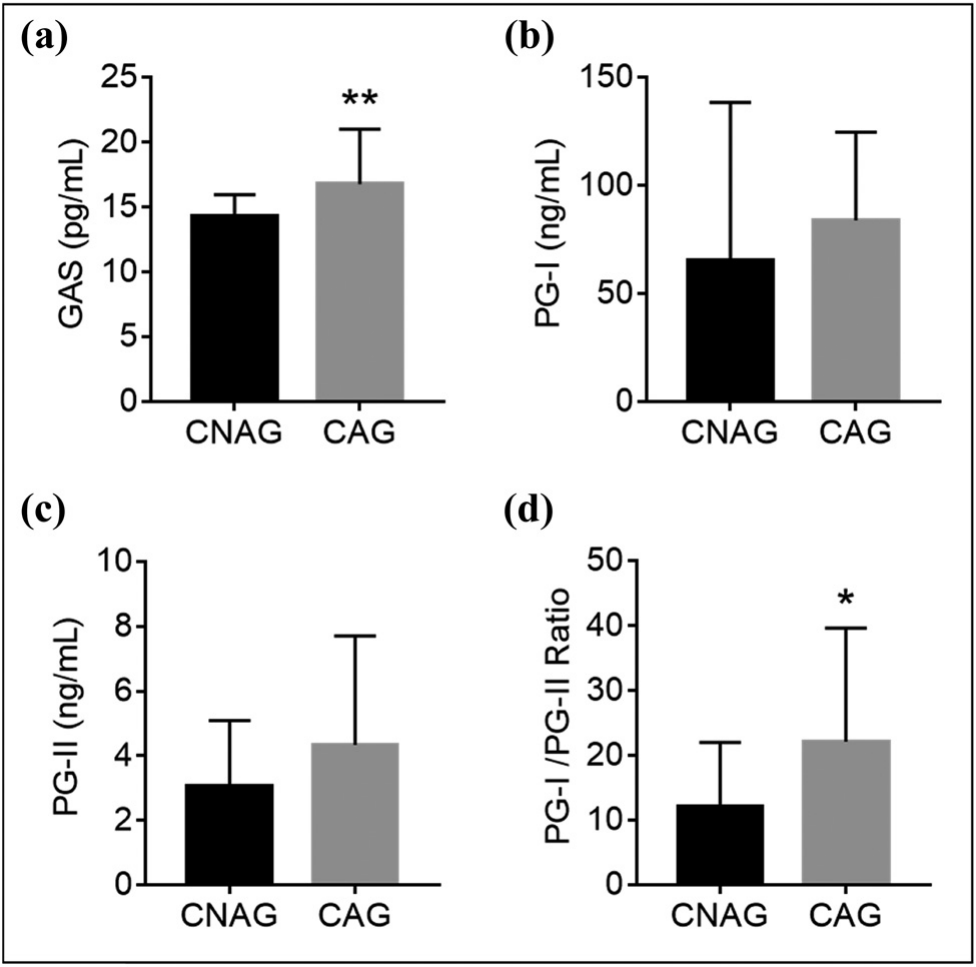
Common serum biomarkers level in CAG patients. (a) Serum GAS level in the CNAG group (*n* = 30) and CAG group (*n* = 30). (b) Serum PGI level in the CNAG group (*n* = 30) and CAG group (*n* = 30). (c) Serum PGII level in the CNAG group (*n* = 30) and CAG group (*n* = 30). (d) Serum PGI/PGII ratio in the CNAG group (*n* = 30) and CAG group (*n* = 30). *p < 0.05 and **p < 0.01.

First, serum PG-I, PG-II, and G-17 concentrations were detected by ELISA assay. Compared with CNAG group, serum GAS level was significantly upregulated in CAG group (Figure 1a). There were no significant difference in serum PG-I, PG-II, or G-17 level between CNAG group and CAG group (Figure 1b and c). However, PG-I/PG-II ratio (PGR) showed a statistically significant difference between CNAG group and CAG group (Figure 1d).

### 3.2 Correlation between serum GAS, PGR, and clinicopathologic factors

Our results further showed that PGR had not significantly correlated with any CAG clinicopathologic factors (Table 1), at least including age, gender, atrophic, intestinal metaplasia, dysplasia, chronic inflammation, and active inflammation. Otherwise, GAS level significantly and positively correlated with atrophy (Table 1).

**Table 1:** Relationships between serum exosomal PGR, GAS, and clinicopathologic factors

Variables		PGR		GAS	
		Mean value ± SD	*P*	Median + QR	*P*
Age	<50 (*n* = 28)	16.35 ± 16.73	0.60	14.34 (13.60, 17.72)	0.42
	≥50 (*n* = 32)	18.39 ± 13.23		16.18 (13.60, 17.54)	
Gender	Male (*n* = 26)	17.30 ± 15.83	0.94	15.26 (13.81, 16.91)	0.94
	Female (*n* = 34)	17.62 ± 13.81		15.38 (13.44, 18.66)	
Atrophic	Absent (*n* = 30)	12.83 ± 10.90	0.05	14.28 (13.44, 15.84)	0.01^*^
	Mild (*n* = 14)	19.00 ± 15.17		17.24 (14.81, 21.85)	
	Moderate (*n* = 11)	22.79 ± 15.75^a^		17.42 (14.68, 20.75)	
	Severe (*n* = 5)	28.96 ± 24.83^*b^		15.49 (14.00, 18.63)	
Intestinal metaplasia	Absent (*n* = 42)	16.98 ± 14.56	0.41	14.51 (13.44, 16.82)	0.08
	Mild (*n* = 9)	13.93 ± 9.39		16.59 (13.52, 18.02)	
	Moderate-severe (*n* = 9)	23.09 ± 20.10		16.77 (15.09, 21.58)	
Dysplasia	Absent (*n* = 52)	16.31 ± 14.76	0.14	14.86 (13.56, 17.59)	0.40
	Light-median (*n* = 8)	24.75 ± 14.32		15.84 (14.85, 18.09)	
Chronic inflammation	Mild (*n* = 31)	14.53 ± 11.70	0.17	14.68 (13.44, 16.290	0.25
	Moderate (*n* = 24)	22.33 ± 19.46		16.13 (13.89, 19.85)	
	Severe (*n* = 5)	15.87 ± 9.06		16.68 (14.03, 20.04)	
Active inflammation	No activity (*n* = 40)	19.03 ± 15.97	0.17	15.84 (13.60, 17.87)	0.77
	Mild (*n* = 17)	12.20 ± 11.91		14.68 (13.60, 16.98)	
	Moderate (*n* = 3)	25.95 ± 4.13		15.37 (13.10, /)	

^a^Moderate vs absent, *P* value is 0.052. ^*b^Severe vs absent, *P* value is 0.023.

### 3.3 Expression correlation between hsa-miR-122-5p and other biomarkers

Serum exosomal hsa-miR-122-5p expression was investigated in our previous study [13]. Here we extracted this result, and then combined with serum GAS, PG-I, and PG-II level to evaluate the relationship between hsa-miR-122-5p and other biomarkers. Our results showed that the expression of hsa-miR-122-5p positively correlated with GAS level, PG-I level, and PG-I/PG-II ratio (PGR), while negatively correlated with PG-II level. However, none of them had significant difference (Table 2).

**Table 2:** Spearman’s correlation analysis between hsa-miR-122-5p and other serum common biomarkers

		GAS	PG-I	PG-II	PGR
hsa-miR-122-5p	Correlation coefficient	0.14	0.16	−0.03	0.06
	Sig. (2-tailed)	0.30	0.22	0.82	0.63

### 3.4 ROC analysis

**Figure 2:**
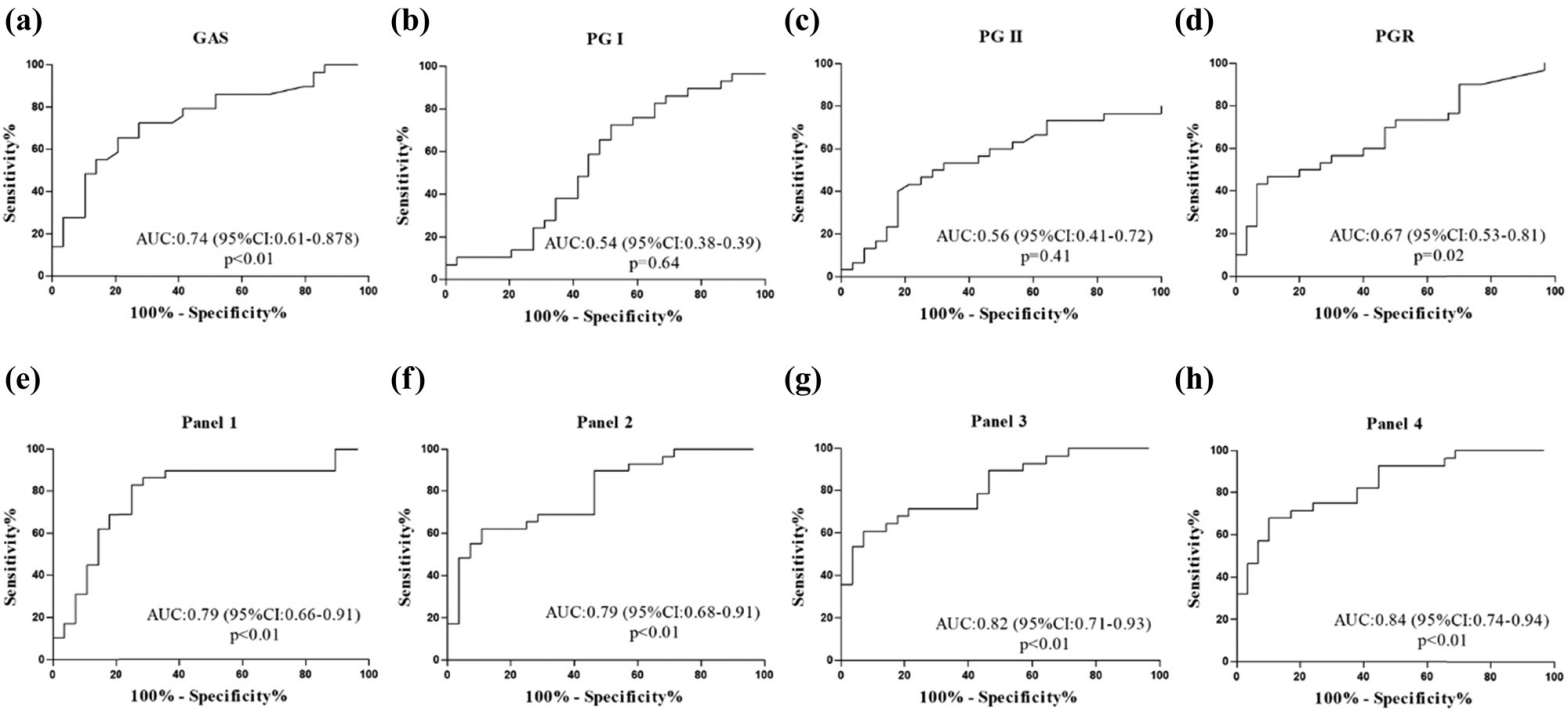
ROC analysis of biomarkers in CAG. (a) ROC analysis of GAS; (b) ROC analysis of PG-I. (c) ROC analysis of PG-II. (d) ROC analysis of PGR. (e) ROC analysis of Panel 1(combination of GAS, PG-I, and PG-II). (f) ROC analysis of Panel 2 (combination of GAS, PG-I, PG-II, and PGR). (g) ROC analysis of Panel 3 (combination of GAS, PG-I, PG-II, PGR, and hsa-miR-122-5p). (h) ROC analysis of Panel 4 (combination of GAS, PGR, and hsa-miR-122-5p).

We also calculated ROC curve of hsa-miR-122-5p in our previous study [13]. Its AUC was 0.76 (95% CI: 0.55–0.96). In the present study, our results showed that AUC of serum GAS, PG-I, PG-II, and PG-I/PG-II ratio (PGR) were 0.74 (95% CI: 0.61–0.88), 0.54 (95% CI: 0.38–0.39), 0.56 (95% CI: 0.41–0.72), and 0.67 (95% CI: 0.53–0.81), respectively (Figure 2a–d). Moreover, AUC of panel 1 (combination of GAS, PG-I, and PG-II) was 0.79 (95% CI: 0.66–0.91) (Figure 2e). AUC of panel 2 (combination of GAS, PG-I, PG-II, and PGR) was 0.79 (95% CI: 0.68–0.91) (Figure 2f). AUC of panel 3 (combination of GAS, PG-I, PG-II, PGR, and hsa-miR-122-5p) was 0.82 (95% CI: 0.71–0.93) (Figure 2g). AUC of panel 4 (combination of GAS, PGR, and hsa-miR-122-5p) was 0.84 (95% CI: 0.74–0.94) (Figure 2h). These results indicated that hsa-miR-122-5p combined with GAS and PGR has a good diagnostic value for CAG disease.

## 4 Discussion

Up to date, the diagnosis of CAG still depends on endoscopic examination and pathological biopsy, which are invasive and thus hard to be accepted by patients. Recently, accumulating studies have discussed the potential value of several serum biomarkers in the non-invasive diagnosis of AG, at least including GAS, PG-I, PG-II, and *Helicobacter pylori* infection [2]. However, these biomarkers usually overlap in diagnosis of gastric cancer, their true sensitivity and specificity need to be further confirmed by multicenter trials. In particular, non-invasive diagnosis of CAG remains largely unknown. In the present study, our results showed that serum GAS level was significantly upregulated in CAG group and significantly correlated to the clinicopathologic factor of CAG, but not serum PG-I or PG-II level. However, there was a significant difference in serum PGR between CNAG group and CAG group. A cross-sectional study highlighted that GAS was upregulated in atrophic gastritis of southwest China, in line with previous studies, they intended to suggest that serum GAS was upregulated in atrophic gastritis patients [14,15]. Otherwise, our results also show that there was no significant difference in PG-I or PG-II between CNAG group and CAG group, but PGR was significantly increased in CAG group. Previous study found that PGR was downregulated in chronic atrophic autoimmune gastritis patients with gastric neuroendocrine tumors [14]. The other study found that pepsinogen level remained unchanged in atrophic antral gastritis patients, while decreased in atrophic fundal gastritis patients [16]. Thus, it needs more evidence to address the relationship between PGR and CAG in future. A cross-sectional study highlighted that GAS was upregulated in atrophic gastritis patients of southwest China.

In the previous study, we compared profiles of serum exosomal miRNA between CAG group and CNAG group. We then upregulated hsa-miR-122-5p significantly in CAG group, and diagnostic value of which (AUC 0.67, 95% CI: 0.52–0.82, sensitivity 62%, and specificity 86%) was better than other significantly changed miRNAs. Moreover, combination of miRNA did not significantly elevate the diagnostic value [13]. We next wonder how to improve the diagnostic value of serum exosomal miRNA in non-invasive diagnosis of CAG. In the present study, our results first showed that there was no significant difference between serum hsa-miR-122-5p expression and serum GAS level, PG-I level, PG-II level, or PGR. Thus, it suggested that hsa-miR-122-5p was an independent biomarker for CAG diagnosis.

We next investigated the possibility of these potential biomarkers on diagnosis of CAG. Previous study found that upregulation of GAS might be a reliable biomarker to predict atrophic gastritis (AUC = 0.92, 95% CI: 0.89–0.94; SE = 85.5%; and SP = 93.2%) [17]. However, the other study showed that GAS was not a good biomarker to predict atrophic gastritis (AUC = 0.58) [15]. Lower PGR might be a reliable biomarker to predict chronic atrophic autoimmune gastritis in patients with gastric neuroendocrine tumors (AUC = 0.92, 95% CI: 0.89–0.94; SE = 85.5%; and SP = 93.2%) [14]. In the present study, our result showed that diagnostic value of GAS (AUC = 0.74, 95% CI: 0.61–0.87; SE = 65.5%; and SP = 79.3%) was better than PG-I, PG-II, or PGR. We further stochastically combined these potential biomarkers to predict CAG, and our result showed that the best panel was consisted of serum exosomal hsa-miR-122-5p, serum GAS, and PGR (AUC = 0.84, 95% CI: 0.74–0.94; SE = 67.9%; and SP = 89.7%).

Taken these together, the present study first discussed the combination of serum exosomal miRNA and other serum biomarkers in diagnosis of CAG, and our results suggested that serum exosomal hsa-miR-122-5p combined with GAS and PGR would elevate the accuracy and specificity in non-invasive screening of CAG. Otherwise, it is true that the number of participants was limited, and it will need more evidence to confirm these findings. However, the present study indeed rationally prompts us to design new clinical studies to further validate the clinical relevance and diagnostic value of these candidate markers in the future study.
